# Nitrogen Source and Rate Management Improve Maize Productivity of Smallholders under Semiarid Climates

**DOI:** 10.3389/fpls.2016.01773

**Published:** 2016-11-30

**Authors:** Amanullah, Asif Iqbal, Ashraf Ali, Shah Fahad, Brajendra Parmar

**Affiliations:** ^1^Department of Agronomy, Faculty of Crop Production Sciences, The University of AgriculturePeshawar, Pakistan; ^2^Crop Physiology and Production Center, College of Plant Science and Technology, Huazhong Agricultural UniversityWuhan, China; ^3^ICAR- Indian Institute of Rice Research, Soil ScienceHyderabad, India

**Keywords:** *Zea mays* L., N levels, N source, yield components, shelling percentage, grain yield, genotypes

## Abstract

Nitrogen is one of the most important factor affecting maize (*Zea mays* L.) yield and income of smallholders under semiarid climates. Field experiments were conducted to investigate the impact of different N-fertilizer sources [urea, calcium ammonium nitrate (CAN), and ammonium sulfate (AS)] and rates (50, 100, 150, and 200 kg ha^−1^) on umber of rows ear^−1^ (NOR ear^−1^), number of seeds row^−1^ (NOS row^−1^), number of seeds ear^−1^ (NOS ear^−1^), number of ears per 100 plants (NOEP 100 plants^−1^), grain yield plant^−1^, stover yield (kg ha^−1^), and shelling percentage (%) of maize genotypes “Local cultivars (Azam and Jalal) vs. hybrid (Pioneer-3025).” The experiment was conducted at the Agronomy Research Farm of the University of Agriculture Peshawar during summers of 2008 (year one) and 2010 (year two). The results revealed that the N treated (rest) plots (the average of all the experimental plots treated with N) had produced higher yield and yield components, and shelling percentage over N-control plots (plots where N was not applied). Application of nitrogen at the higher rate increased yield and yield components in maize (200 > 150 > 100 > 50 kg N ha^−1^). Application of AS and CAN had more beneficial impact on yield and yield components of maize as compared to urea (AS > CAN > urea). Hybrid maize (P-3025) produced significantly higher yield and yield components as well as higher shelling percentage than the two local cultivars (P-3025 > Jalal = Azam). Application of ammonium sulfate at the rate of 200 kg N ha^−1^ to hybrid maize was found most beneficial in terms of higher productivity and grower's income in the study area. For the two local cultivars, application of 150 kg N ha^−1^ was found more beneficial over 120 kg N ha^−1^ (recommended N rate) in terms of greater productivity and growers income.

## Introduction

Maize (*Zea mays* L.) is the 2nd important crop after wheat in the Khyber Pakhtunkhwa Province of Pakistan. The yield of maize is very low (1868 kg ha^−1^) in Khyber Pakhtunkhwa (semiarid climate) than the average yield (3990 kg ha^−1^) in the country (Amanullah et al., 2009a,b; MINFAL, 2012). The reasons for low crop productivity in semiarid climate are: (1) low soil moisture availability (2) low soil fertility (Amanullah et al., 2012), and (3) indiscriminate use of chemical fertilizers by smallholders (Amanullah et al., 2015a). According to Oad et al. (2004) and Amanullah and Khan (2015), the major problems in the way of increasing yield at farmer's fields are the inappropriate nutrients supply. Among the plant nutrients, nitrogen (N) management is one of the most important factor required for improving crop productivity and profitability under semiarid climates (Amanullah, 2016). According to Guo et al. (2016), N is the most yield-restraining nutrient in crop production globally. Our previous research work on maize and nitrogen management revealed that proper nitrogen rates and nitrogen timings (splits) management improve maize growth and development (Amanullah et al., 2007, 2008, 2009a,b; Amanullah and Shah, 2010), yield and yield components (Amanullah et al., 2009a), grain quality (Amanullah et al., 2010), and growers income (Amanullah et al., 2010) in the study area. In another study (Amanullah et al., 2015b), we noticed that the integrated use of nitrogen (120 or 150 kg N ha^−1^) along with compost (2 t ha^−1^) improved yield and yield components in maize under deep (45 cm) than shallow (15 cm) tillage system under semiarid climate.

Increasing human population in Pakistan is resulting in less land for cultivation. Therefore, increase in crop productivity depends on higher yield per unit area. Increased cropping intensity especially under cereal based cropping system removes plant nutrients (Amanullah and Inamullah, 2016) from the soil, in addition to natural nitrogen losses (Gehl et al., 2005). Therefore, it has become of foremost importance to select high yielding varieties and genotypes that can uptake and utilize nitrogen more efficiently (Hirel et al., 2001). Efficient nitrogen fertilizer management is essential for achieving economic yields and for enhancing N use efficiency (Pan et al., 2012; Yousaf et al., 2014). Selection of high yielding cultivars is important for food security and growers incomes (Geiger, 2009). Best nitrogen management practices play an important role in increasing crop productivity (Fageria et al., 2006). Best nitrogen management practices minimize N losses and increases N availability for crops which increase nitrogen use efficiency and reduce negative impacts of N on the environment (Havlin et al., 2009). According to Amanullah (2014), N use efficiency (NUE) decrease with increase in N rate and the NUE was higher with application of ammonium sulfate (AS) over calcium ammonium nitrate (CAN) and urea. The hybrid maize had higher NUE than the local cultivars (Pioneer-3025 > Jalal > Azam). Ammonium sulfate increases NUE, however, its higher cost make it less profitable N-fertilizer for the maize growers in Northwest Pakistan (Amanullah et al., 2015a). The decline in the price of AS could make it more profitable and most suitable N-fertilizer source under semiarid climates in terms of higher NUE (Amanullah, 2014), higher net returns (Amanullah et al., 2015b), and better seed quality (Khan, 2016).

In the semiarid climates, growing maize hybrids are more efficient than local cultivars in terms of higher NUE and grower's income. However, no previous research was reported to investigate the response of different maize genotypes to different N sources × N levels interaction. This experiment was conducted with an overall objective to find the best level and best source of N fertilizer for improving yield and yield components of maize genotypes. Sustainable crop production relies on the continuous renewal of soil fertility through a balance between N demand and supply in cropping systems (Yousaf et al., 2016). Better management of high yielding crops with lower N loss is desperately needed to achieve sustainable agriculture (Duan et al., 2014). The current research project was therefore conducted to investigate yield and yield components response of maize genotypes [Pioneer-3025 (hybrid) in comparison with local cultivars (Jalal and Azam)] to N rates and sources to find out best N source and their level for higher maize productivity in the study area.

## Materials and methods

### Site description

“Field experiment was conducted at the Agronomy Research Farm of the University of Agriculture Peshawar for two years during summers of 2008 (year one) and 2010 (year two). The experimental farm is located at 34.01° N latitude, 71.35° E longitude at an altitude of 350 m above sea level in Peshawar valley. Peshawar is located about 1600 km north of the Indian Ocean and has continental type of climate. The research farm is irrigated by Warsak canal from river Kabul. Soil texture is clay loam, low in organic matter (0.87 %), extractable phosphorus (6.57 mg kg^−1^), exchangeable potassium (121 mg kg^−1^), and alkaline (pH 8.2) and is calcareous in nature (Amanullah et al., 2009a).” The climate of Peshawar is semiarid where the mean annual rainfall is very low (300–500 mm), 60–70% rainfall occurs in summer, while the remaining 30–40% rainfall occurs in winter (Amanullah et al., 2010a). Weather data for the maize growing periods in both years are given in Table 1.

**Table 1:** **Weather data of maize growing periods in 2008 and 2010 at Peshawar, Pakistan**.

**Weather data**	**Growing season 2008**				**Growing season 2010**			
	**July**	**August**	**Sept**.	**Oct**.	**July**	**August**	**Sept**.	**Oct**.
Mean temperature °C	31	30	28	25	31	29	24	24
Max temperature °C	36	35	34	32	34	33	34	32
Min temperature °C	26	25	22	19	26	26	21	19
Precipitation (mm)	37	274	38	1	409	125	4	0
Mean humidity (%)	66	71	63	60	75	80	63	65
Wind speed (km/h)	19	14	11	6	15	13	11	5

### Experimentation

“A field experiment was conducted in a randomized complete block design with split-plot arrangement using three replications. Factorial experimental treatments were four N rates [50, 100, 150, and 200 kg N ha^−1^] and three N-fertilizer sources (Urea having 46% N, Calcium Ammonium Nitrate having 26% N and 10% Ca, and Ammonium having 21% N and 24% S) used as main plots factor, while three maize genotypes (Jalal, Azam, and Pioneer-3025) were used as sub plots factor. One control plot (N not applied) was also used in each replication as check for comparison. A sub-plot size of 4.2 m by 5 m, having 6 rows, 5 m long, and 70 cm apart was used. A uniform basal dose of 60 kg P ha^−1^ as single super phosphate (18% P_2_O_5_), and 60 kg K ha^−1^ as sulfate of potash (50% K_2_O) was applied and mixed with the soil during seedbed preparation (Amanullah et al., 2015a).” Nitrogen in the form of urea, CAN, and AS was applied in two equal splits i.e., 50% at sowing and 50% at 2nd irrigation (30 days after emergence). “Data were recorded on number of rows ear^−1^, number of seed row^−1^, number of seed ear^−1^, number of ears 100 plants^−1^, grain yield, stover yield, and shelling percentage. Data on number of rows ear^−1^ was calculated by counting number of rows in ten selected ears and then it was averaged. Data on number of seed rows per ear was calculated by counting seed rows in ten selected ears and then it was averaged. Number of seeds ear^−1^ was calculated on ten randomly selected ears from each plot and then average was worked out. Data on number of ears 100 plants^−1^ was calculated by counting number of ears in 100 plants and then it was averaged (Amanullah and Khan, 2015).” Three central rows of each treatment was harvested the material was dried and converted into biological yield (kg ha^−1^). The ears of the three central rows were removed, threshed, grains were cleaned and weighted and then converted into grain yield (g plant^−1^). Stover was determined as the difference between biological minus grain yield, and shelling percentage each treatment was determined using the following formulae:

$$\begin{array}{l} Stover\;yield\;(kg\;ha^{-1})=Biological\;yield\;(kg\;ha^{-1})\\ \;-\;Grain\;yield\;(kg\;ha^{-1})\\ \;Shelling\;percentage\;=\frac{Grains\;weight\;of\;10\;ears}{Total\;weight\;of\;10\;ears}\;\times \;100 \end{array}$$

### Statistical analysis

Data were subjected to analysis of variance (see Table 2 for details) according to the methods described by Steel et al. (1997), and means between treatments was compared by least significant difference (*P* ≤ 0.05). The following model was used for the statistical analysis of data:

$$\begin{array}{l} Y=u+N+S+G+C\;v\;R+N\;x\;S+N\;x\;G+S\;x\;G\\ \;+\;N\;x\;S\;x\;G+E \end{array}$$

Where: *Y*, independent variable; *u*, overall mean; *N*, Nitrogen rates; *S*, Nitrogen sources; *G*, Genotypes; *C v R*, Control vs. Rest; *E*, random or pooled error.

**Table 2:** **Sample analysis of variance used for the statistical analysis of the data recorded in both years**.

**Source of variance**	**Degrees of freedom**
Replications (03)	02
Treatments (01 control + 03 N levels × 04 N sources = 13)	[12]
Nitrogen Levels (03)	(02)
Nitrogen Sources (04)	(03)
Nitrogen Levels × Nitrogen Sources (3 × 4)	(06)
Control vs. rest (control = no N applied & rest = average of N treated plots)	(01)
**Error I**	24
Genotypes (03)	02
Treatments × Genotypes (12 × 02)	[24]
(Control vs. rest) × Genotypes (01 × 02)	(02)
Nitrogen Levels × Genotypes (02 × 02)	(04)
Nitrogen Sources × Genotypes (03 × 02)	(06)
Nitrogen Levels × Nitrogen Sources × Genotypes (02 × 03 × 02)	(12)
**Error II**	52
Total (03 × 13 × 03 – 01 = 117 – 01)	116

Where: The degree of freedom in () is the further splits of degree of freedom in [] viz. [12] and [24], respectively.

## Results

**Figure 1:**
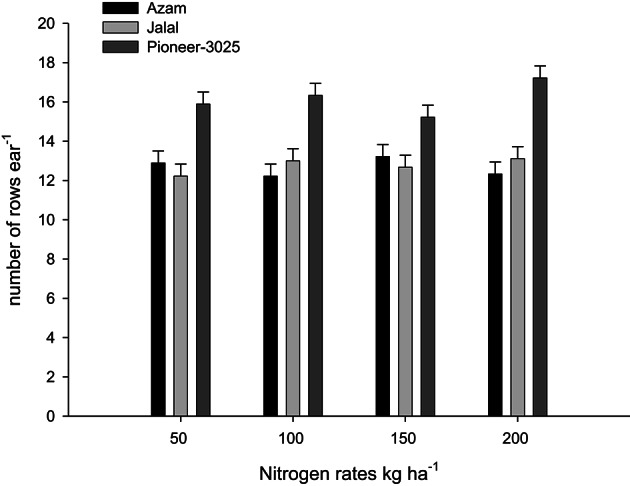
**Nitrogen rates and genotypes interaction influence number of rows ear^−1^ in maize during 2008**.

**Figure 2:**
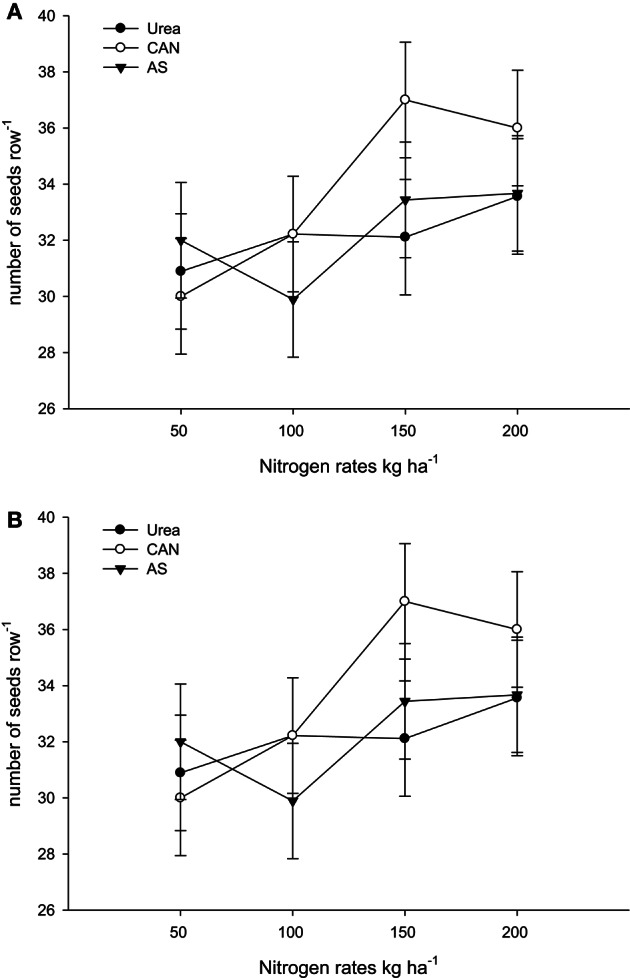
**(A)** Nitrogen rates and sources interaction influence number of seeds row^−1^ in maize during 2008. **(B)** Nitrogen rates and sources interaction influence number of seeds row^−1^ in maize during 2010.

**Figure 3:**
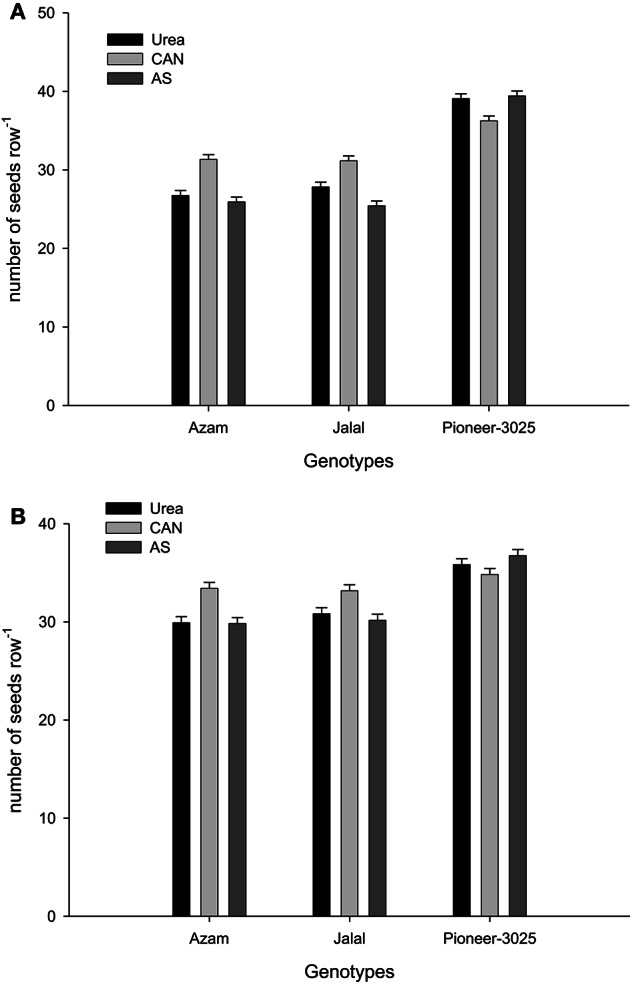
**(A)** Nitrogen sources and genotypes interaction influence number of seeds row^−1^ in maize during 2008. **(B)** Nitrogen sources and genotypes interaction influence number of seeds row^−1^ in maize during 2010.

**Figure 4:**
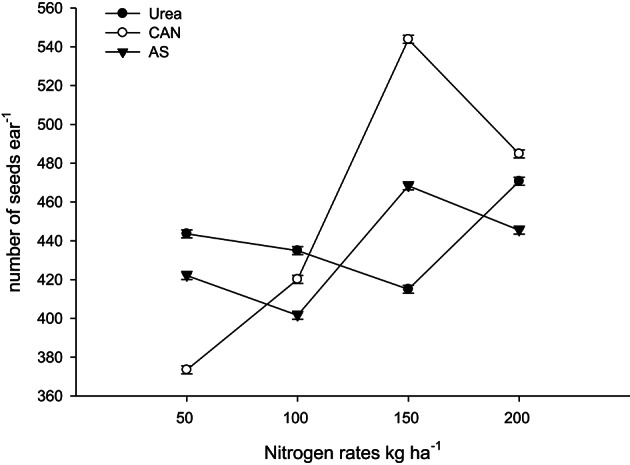
**Nitrogen rates and sources interaction influence number of seeds ear^−1^ in maize during 2008**.

**Figure 5:**
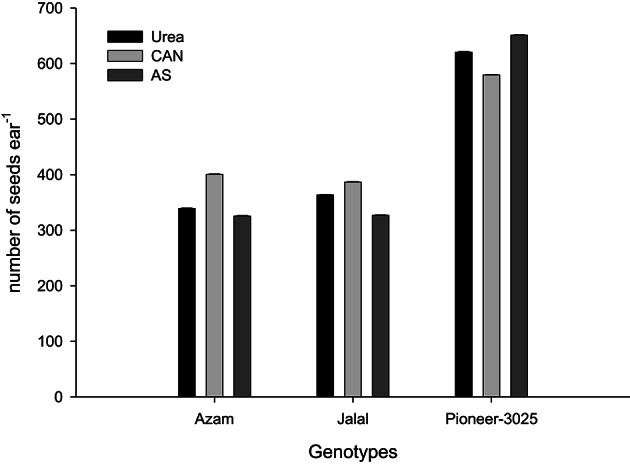
**Nitrogen sources and genotypes interaction influence number of seeds ear^−1^ in maize during 2008**.

**Figure 6:**
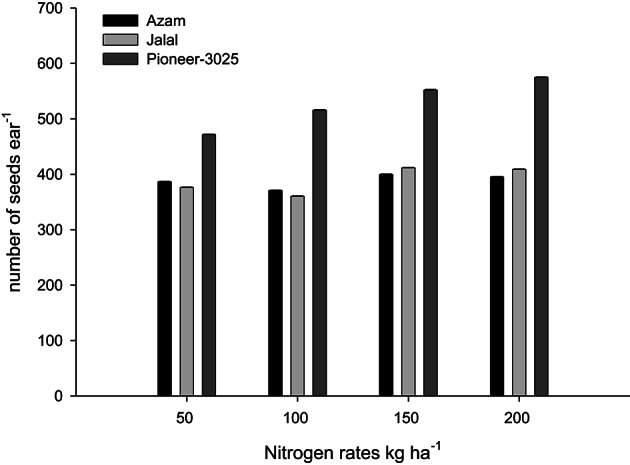
**Nitrogen rates and genotypes interaction influence number of seeds ear^−1^ in maize during 2010**.

It is clear from Table 3, that the rest (N treated plots) had produced number of rows ear^−1^ (NOR ear^−1^), number of seeds row^−1^ (NOS row^−1^), number of seeds ear^−1^ (NOS ear^−1^) than control plots (N not applied). In both the years, NOR ear^−1^, NOS row^−1^, and NOS ear^−1^ increased significantly (*P* ≤ 0.05) with application of N in two higher than the two lower rates. Application of AS (ammonium sulfate) produced more NOR ear^−1^ than urea and CAN (calcium ammonium nitrate). On the other hand, application of CAN produced more NOS row^−1^ and NOS ear^−1^ as compared to urea (Table 3). The hybrid maize had produced higher NOR ear^−1^, NOS row^−1^, and NOS ear^−1^ than the two local cultivars in both the years (Table 3). Increase in N level produced more NOR ear^−1^ and the increase was more in the maize hybrid than the two local cultivars that resulting in significant N-level × genotypes (N × G) interaction (Figure 1). Interaction of nitrogen source and nitrogen rates (N × S) in year one (Figure 2A) and year two (Figure 2B) indicating that the increase in N level had increased number of seeds per row while using different N sources. Interaction of N source and genotypes (S × G) indicated that in both years i.e., year one and two (Figures 3A,B, respectively), maize hybrid performed better by producing more number of seeds per row with each N source as compared with the two local maize cultivars. Interaction of N source and rates (N × S) had significant effects on NOS ear^−1^ (Figure 4), indicating that the NOS ear^−1^ increased to maximum with CAN applied at the rate of 150 kg N ha^−1^. While in case of urea the NOS ear^−1^ increased to maximum with the highest rate of 200 kg N ha^−1^ (Figure 4). Application of AS at the rate of 150 kg N ha^−1^ increased NOS ear^−1^ in both years. The NOS ear^−1^ increased to maximum in hybrid maize with application of CAN as compared to other N sources resulting in significant S × G interaction (Figure 5). The interaction between N rates and genotypes (N × G) indicated that NOS ear^−1^ increased in hybrid maize at all four levels of N and increased to maximum at the highest rate of 200 kg N ha^−1^ (Figure 6). On the other hand, the NOS ear^−1^ in the local cultivars increased up to 150 kg N ha^−1^ and further increase in N level had not significantly increased NOS ear^−1^.

**Table 3:** **Control (N not applied) vs. rest (all N applied plots), N rate, N source and genotypes influence on NOR Ear^−1^, NOS row^−1^, and NOS Ear^−1^of maize (mean effects)**.

**Treatments**	**Year one**			**Year two**		
**N rate (kg ha^−1^)**	**NOR Ear^−1^**	**NOS row^−1^**	**NOS Ear^−1^**	**NOR Ear^−1^**	**NOS row^−1^**	**NOS Ear^−1^**
50	13.7	29.6	413.0	13.3	30.9	411.3
100	13.8	29.7	418.8	13.1	31.4	415.5
150	13.7	34.3	475.7	13.3	34.2	454.4
200	14.2	32.0	466.9	13.3	34.1	459.5
LSD (*P* ≤ 0.05)	ns	2.4	41.7	ns	1.5	34.8
**N FERTILIZER SOURCE**						
Urea	13.9	31.2	441.0	13.4	32.2	434.5
CAN	13.7	32.9	455.5	12.9	33.8	437.2
AS	13.9	30.2	434.3	13.3	32.2	433.8
LSD (*P* ≤ 0.05)	ns	2.1	ns	ns	1.30	ns
**MAIZE GENOTYPES**						
Azam	12.7	28.0	355.0	12.5	31.0	387.9
Jalal	12.7	28.1	358.8	12.4	31.4	389.1
Pioneer-3025	16.2	38.2	617.0	14.7	35.8	528.5
LSD (*P* ≤ 0.05)	0.6	1.9	33.2	0.5	1.20	19.9
Control	12.9	27.2	351.8	12.2	28.3	346.0
Rest	13.8	31.4	443.6	13.2	32.7	435.2
**INTERACTIONS**						
N × S	ns	^*^(Figure 2A)	^*^(Figure 4)	ns	^*^(Figure 2B)	ns
S × G	ns	^*^(Figure 3A)	^*^(Figure 5)	ns	^*^(Figure 3B)	ns
N × G	^*^(Figure 1)	ns	ns	ns	ns	^*^(Figure 6)
S × N × G	ns	ns	ns	ns	ns	ns

Where: NOR Ear^−1^, number of rows per ear; NOS row^−1^, number of seeds per row; NOS Ear^−1^, number of seeds per ear. ns stands for non-significant data while

* *stands for significant data at 5% level of probability*.

**Figure 7:**
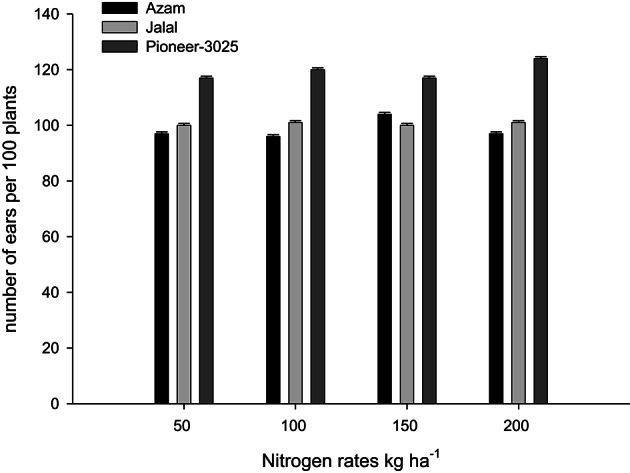
**Nitrogen rates and genotypes interaction influence number of ears per 100 plants in maize during 2010**.

**Figure 8:**
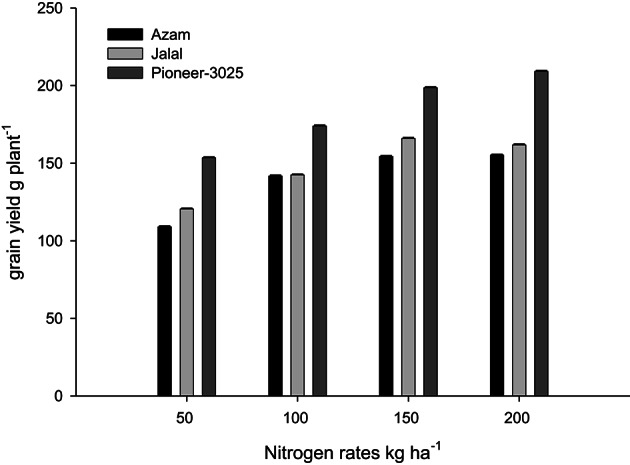
**Nitrogen rates and genotypes interaction influence grain yield of maize during 2010**.

**Figure 9:**
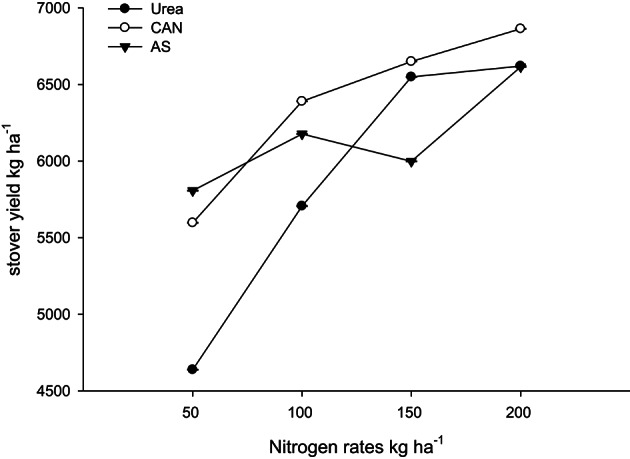
**Nitrogen rates and sources interaction influence stover yield of maize during 2008**.

**Figure 10:**
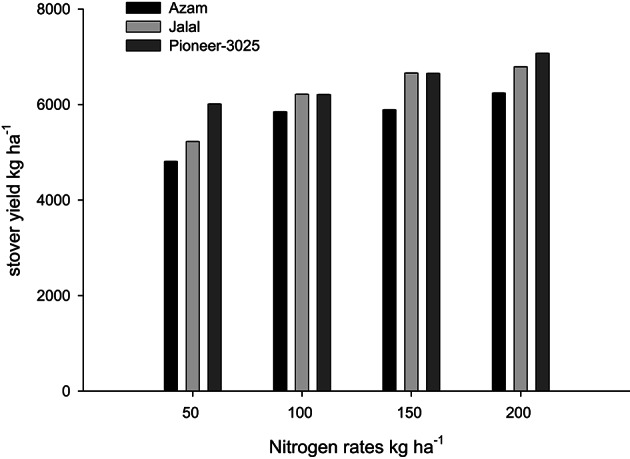
**Nitrogen rates and genotypes interaction influence stover yield of maize during 2008**.

Likewise, it is clear from Table 4 that the N rest had produced more number of ears per 100 plants (NOEP 100 plants), grain yield, stover yield, and shelling percentage than the control plots. In both years NOEP 100 plants, grain and stover yields (Table 4) increased significantly with application of N in two higher than the two lower rates. The effect of nitrogen sources was not significant for NOEP 100 plants (Table 4). Ammonium sulfate treated plots produced higher grain yield, followed by CAN, while less grain yield was recorded from urea (Table 4). Application of CAN produced more stover yield, followed by AS, while less stover yield was recorded from urea treated plots. The hybrid maize had produced higher NOEP 100 plants, grain, and stover yields than the two local cultivars in the two years (Table 4). Interaction between N rates and genotypes (N × G) had significant effects on NOEP 100 plants (Figure 7). The NOEP 100 plants increased in maize hybrid while increasing N level up to 200 kg N ha^−1^, while in the local cultivars NOEP 100 plants increased up to 150 kg N ha^−1^ and further increase in N had no significant effect on NOEP 100 plants. Interaction between N rates and genotypes (N × G) had significant effects on grain yield in 2nd year (Figure 8). The grain yield increased in maize hybrid while increasing N rate as compared to the two local maize cultivars. The grain yield of the two cultivars increased with increase in N up to 150 kg N ha^−1^ and further increase in N rate 200 kg N ha^−1^ did not increased grain yield significantly. Interaction between N source and N rates (N × S) had significant effects on stover yield in the first year only (Figure 9). The stover yield increased with increase in N rates while using each N fertilizer source. Interaction between N rates and genotypes (N × G) indicated that stover yield increased in hybrid maize with increase in N level up to 200 kg N ha^−1^, while in the local cultivars stover yield increased up to 150 kg N ha^−1^ and further increase in N up to 200 kg N ha^−1^ did not increased the stover yield significantly (Figure 10).

**Table 4:** **Control (N not applied) vs. rest (all N applied plots), N rate, N source and genotypes influence on NOEP 100 plants, grain yield g plant^−1^, stover yield kg ha^−1^, and shelling percentage (%) of maize (mean effects)**.

**Treatments**	**Year one**				**Year two**			
**N rate (kg ha^−1^)**	**NOEP 100 plants**	**GY g plant^−1^**	**SY kg ha^−1^**	**SP (%)**	**NOEP 100 plants**	**GY g plant^−1^**	**SY kg ha^−1^**	**SP (%)**
50	92	97.6	5347	79.9	105	127.6	5994	77.4
100	95	102.0	6091	80.6	106	152.6	6435	78.6
150	96	115.7	6399	80.7	107	172.9	7438	78.7
200	96	113.0	6700	81.3	107	175.3	7868	79.1
LSD (*P* ≤ 0.05)	2	5.2	330	ns	ns	5.8	928	ns
**N FERTILIZER SOURCE**								
Urea	94	99.8	5878	80.1	105	150.2	6697	77.9
CAN	95	107.5	6375	80.3	107	157.6	7387	78.4
AS	96	113.9	6149	81.4	107	163.5	6717	79.2
LSD (*P* ≤ 0.05)	ns	4.5	286	ns	ns	5.1	ns	ns
**GENOTYPES**								
Azam	87	90.9	5696	81.2	99	140.0	6215	79.5
Jalal	92	98.4	6222	80.6	101	147.6	7055	78.1
Pioneer-3025	106	131.9	6485	80.1	119	183.7	7531	77.8
LSD (*P* ≤ 0.05)	1.827	4.5	168	ns	2.91	4.9	594	ns
Control	89	76.4	4177	79.8	95	96.6	5520	77.2
Rest	95	107.1	6134	80.6	106	157.1	6934	78.5
**INTERACTIONS**								
N × S	ns	ns	^*^(Figure 7)	ns	ns	ns	ns	ns
S × G	ns	ns	ns	ns	ns	ns	ns	ns
N × G	ns	ns	^*^(Figure 8)	ns	^*^(Figure 9)	^*^(Figure 10)	ns	ns
S × N × G	ns	ns	ns	ns	ns	ns	ns	ns

Where: NOEP 100 plants, number of ear per 100 plants; GY, grain yield; SY, stover yield; SP, shelling percentage. ns stands for non-significant data while

* *stands for significant data at 5% level of probability*.

## Discussion

### Nitrogen rate

The higher in yield and yield components of maize under higher N rates over the lower N rates was attributed to the increase in leaf area index (LAI) and total dry matter (TDM) accumulation (Amanullah et al., 2014), increase in NUE and harvest index (Amanullah, 2014). Hejazi and Soleymani (2014) reported that the N control treatment had the lowest yield components and grain yield than the N fertilized plots. Increase in yield components of maize in the N fertilized probably may be attributed to less competition among maize plants for nitrogen, and the plants therefore accumulated more biomass and partitioned more dry matter into the reproductive parts (ears) because of its higher LAI (Amanullah et al., 2014). The higher capacity of the plants to convert more photosynthates into the reproductive parts increases maize productivity. Increase in maize productivity with nitrogen fertilization was also reported by many workers (Malaiya et al., 2004; Amanullah et al., 2009a, 2015b). The increase in yield and yield components in the current study with N supply probably may be due to the increase in light interception (Amanullah et al., 2008) because of it higher LAI (Amanullah et al., 2014). Many researchers reported improvement in maize yield and yield components while increasing N level. For example, Malaiya et al. (2004) reported that N fertilizer treatments produced higher number of ears in maize crop. Zeidan et al. (2006) reported that grains number ear^−1^ in maize was maximum at the highest nitrogen rate. Alizade et al. (2007) reported increase in yield components with higher N level. Khatun et al. (2012) and Sampath et al. (2013) revealed that stover yield in maize increases with increasing N rate. According to Yousaf et al. (2016), for a sustainable crop production continuous renewal of soil fertility through a balance between N demand and supply in cropping systems is important. Guo et al. (2016) reported that N is the most yield-restraining nutrient in crop production globally.

### Nitrogen source

Yield and yield components in maize increased with application of either AS or CAN as compared with urea. The increase in yield and yield components with AS or CAN over urea may be attributed to the increase in LAI and total dry matter accumulation (Amanullah et al., 2014) and increase in N-use efficiency (Amanullah, 2014). According to the review of Chien et al. (2011), ammonium sulfate is the best N-fertilizer source which contains frees sulfur and had many potential agronomic and environmental benefits over urea and ammonium nitrate. However, because of its (AS) highest N cost than the N cost of other N sources (CAN and urea), the small landholder can't afford to use AS for crop production (Amanullah et al., 2014). The higher transportation charges of AS is also more than urea and CAN (Amanullah et al., 2014). On the other hand, the low hygro-scopicity of AS and as a source of both N (21%) and S (24%), and its strongly acid reaction in the soil is the advantage of AS on high pH soils especially on S-deficient soils in semiarid climate (Havlin et al., 2009). According to Sas (2006), CAN is an important N-fertilizer source for neutralizing soil acidity. Amanullah et al. (2013) found significant differences among the three N-sources (Urea, CAN, and AS) when these fertilizers were applied in the form of foliar spray. According to Fageria et al. (2011), the higher and lower N rate in the form of AS increase yield, while the intermediate N rates (125–275 mg N kg^−1^) in the form of urea was better than AS.

### Genotypes

The supremacy of hybrid maize in terms of higher productivity over the two local cultivars was attributed to the higher LAI and total dry matter accumulated by the hybrid maize than the local cultivars (Amanullah et al., 2014). Moreover, the higher NUE of hybrid maize (Amanullah, 2014) could also be the possible cause of higher yield and yield components produced by the maize hybrid over the two local cultivars. Hokmalipour et al. (2010) reported significant variation in N use efficiency, productivity and growers income among various maize cultivars. Many other workers (e.g., Akram et al., 2010; Sampath et al., 2013; Hejazi and Soleymani, 2014; Sani et al., 2014) reported variations in yield and yield components among different maize cultivars. Cui et al. (2009) reported that the interaction between genotypes and nitrogen significantly influence grain yield and NUE in maize.

## Conclusions

Nitrogen management is one of the most important factor for improving crop productivity under semiarid climates. The results of this study confirmed the existence of significant variability in yield and yield components of maize depending on genotypes inheritance, differences in N fertilizer source and rates as well as differences in the weather condition. Application of nitrogen at the rate of 150 and 200 kg N ha^−1^ in the form of urea, 100 and 150 kg N ha^−1^ in the form of CAN (calcium ammonium nitrate) and 50 and 100 kg N ha^−1^ in the form of ammonium sulfate was more beneficial for improving maize productivity in the study area. The current higher price of fertilizers along and the less purchasing power had negative impact on smallholder's income. The superiority of AS over urea probably may be due to the presence of sulfur (24%) which is lacking in urea. Ammonium sulfate has an acidifying effect on soil, therefore, its continuous use may be advantageous on alkaline soils especially under arid and semiarid climates. The superiority of CAN over urea may be attributed to the availability of N in the two different forms (ammonium and nitrate) which make the N availability for long time than urea. Decrease in the cost of hybrid seeds and N-fertilizers especially ammonium sulfate will decrease production cost and could increase crop productivity, profitability and sustainability under semiarid climates.

## Author contributions

Amanullah designed the research project, analyzed the data, and wrote the manuscript. AI and AA performed the experiments, collected the data, made tables and figures. SF and BP helped in the editing of the manuscript.

## Funding

This research was supported by The University of Agriculture Peshawar from its endowment fund.

### Conflict of interest statement

The authors declare that the research was conducted in the absence of any commercial or financial relationships that could be construed as a potential conflict of interest.
